# Welcome to *Epigenetics & Chromatin*

**DOI:** 10.1186/1756-8935-1-1

**Published:** 2008-10-27

**Authors:** Steven Henikoff, Frank Grosveld

**Affiliations:** 1 Basic Sciences Division and Howard Hughes Medical Institute, Fred Hutchinson Cancer Research Center, Seattle, Washington, USA; 2 Department of Cell Biology, and Department of Reproduction and Development, Erasmus MC, University Medical Center, Rotterdam, The Netherlands

## 

We are happy to introduce the new journal *Epigenetics & Chromatin*, which finds its origin in the EU Network of Excellence 'the Epigenome' [1] and the positive response by colleagues around the world who have participated in this venture.

We often hear the terms 'epigenetic' and 'chromatin' used interchangeably in the context of inheritance that is not encoded in DNA sequence; however, these two terms have very different histories. The concept of epigenesis was introduced by Aristotle as the unfolding of a developmental program to form an animal or plant from an amorphous egg or spore through differentiation as opposed to the view that the new complex organism is already present in the egg or spore. Much later, Conrad Waddington introduced the more modern term epigenetics by hypothesizing the existence of an epigenetic landscape that channels gene activity to maintain stable inheritance during development [2]. The term chromatin was used by Walther Flemming in the 1880s for what later were called mitotic chromosomes [3], but eventually its meaning shifted, referring instead to the complex of DNA and protein that chromosomes are made of. Although it has long been understood that chromatin is involved in maintaining the epigenetic landscape, just how this happens had long remained mysterious, despite spectacular progress in understanding basic genetic mechanisms. Only in recent years have connections between epigenetics and chromatin been explored at the molecular level, thanks in large part to rapidly improving technologies.

As epigenetics and chromatin have converged into a single field of research, it has become increasingly important that researchers at all levels learn about advances made in areas that they might be otherwise unfamiliar with. For example, beginning with the first Epigenetics Gordon Conference [4], which was held in 1995, a concerted effort has been made to invite speakers and attract participants who study epigenetic processes in plant, animal and fungal systems. The wisdom of this strategy is clear in hindsight, insofar as some of the most important advances in the epigenetics and chromatin field have come from unexpected biological systems, such as the role of small RNAs in gene silencing in plants, worms and fission yeast. We do not know where the next breakthroughs will originate, so in launching *Epigenetics & Chromatin *we aim for high-quality submissions in diverse biological systems that cover a wide range of topics.

One might ask whether a new journal that is dedicated to this subject is needed. For example, there are already many journals devoted to the traditional study of development, a discipline that chromatin studies have had a large impact on in recent years. However, if we ask how many articles have been published over the past decade categorized as being concerned with development (based on Medical Subject Headings found in PubMed) we find that the number has only doubled, while the number of articles on epigenetics has increased 15-fold. Whereas 10 years ago there were almost three times as many articles on the subject of development as on the subject of epigenetics, the ratio has now reversed. Meanwhile, articles categorized as being concerned with both epigenetics and chromatin have increased more than 20-fold over the past decade. This disproportionate expansion of epigenetics and chromatin as a field relative to traditional disciplines is also evident from the increasing number of conferences dedicated to the subject, and the trend for traditional meetings on related subjects, such as transcription, to more strongly emphasize epigenetics and chromatin.

*Epigenetics & Chromatin *will publish articles aimed at understanding how gene and chromosomal elements are regulated and their activities maintained during cell division, self-renewal, differentiation and environmental alteration. Epigenetic research encompasses studies that use model systems to discover and investigate epigenetic mechanisms, as well as studies aimed at combating diseases that involve epigenetic processes. Topics include, but are not limited to, gene activation, silencing and imprinting, cellular reprogramming, nucleosome modification, assembly and remodeling, DNA methylation, chromatin structure and dynamics, chromosomal maintenance elements, dosage compensation, intra- and inter-chromosomal interactions and prion inheritance. Approaches that apply cutting-edge technologies to problems in the field are especially welcome. We will also be soliciting reviews in emerging areas of research based on suggestions from members of our editorial board [5], whose expertise spans a wide range of disciplines.

Manuscripts submitted to *Epigenetics & Chromatin *will be reviewed by internationally recognized experts in the field of epigenetics and chromatin, selected in part from our editorial board. Reviews will be rapid and the suitability of a manuscript for publication will be assessed solely on criteria of scientific excellence. Upon acceptance, articles will be made immediately available online with a fixed-article processing charge without constraints of page limitations or color page charges. Online open-access publication means that all articles will be available to all readers without charge [6] and that movies and animations are only a click away.

The field of epigenetics is at an exciting stage, one that seems comparable to the field of genetics before the elucidation of the structure of DNA and the genetic code. We know most of the parts, but we are still unclear as to how they work together as a system to faithfully maintain gene expression states. As our understanding of epigenetic inheritance and chromatin-based interactions progresses and the terms epigenetics and chromatin become more precisely defined, we expect that *Epigenetics & Chromatin *will evolve to accommodate the latest advances. The editors are committed to making this new venture a success, and we hope that you share our vision.

## Competing interests

The authors declare that they have no competing interests.
